# Stress affects instrumental learning based on positive or negative reinforcement in interaction with personality in domestic horses

**DOI:** 10.1371/journal.pone.0170783

**Published:** 2017-05-05

**Authors:** Mathilde Valenchon, Frédéric Lévy, Chantal Moussu, Léa Lansade

**Affiliations:** 1 INRA, Centre Val de Loire, UMR85 Physiologie de la Reproduction et des Comportements, Nouzilly, France; 2 CNRS, UMR6175 Physiologie de la Reproduction et des Comportements, Nouzilly, France; 3 Université François Rabelais, Tours, France; 4 IFCE, Nouzilly, France; University of Lethbridge, CANADA

## Abstract

The present study investigated how stress affects instrumental learning performance in horses (*Equus caballus*) depending on the type of reinforcement. Horses were assigned to four groups (*N* = 15 per group); each group received training with negative or positive reinforcement in the presence or absence of stressors unrelated to the learning task. The instrumental learning task consisted of the horse entering one of two compartments at the appearance of a visual signal given by the experimenter. In the absence of stressors unrelated to the task, learning performance did not differ between negative and positive reinforcements. The presence of stressors unrelated to the task (exposure to novel and sudden stimuli) impaired learning performance. Interestingly, this learning deficit was smaller when the negative reinforcement was used. The negative reinforcement, considered as a stressor related to the task, could have counterbalanced the impact of the extrinsic stressor by focusing attention toward the learning task. In addition, learning performance appears to differ between certain dimensions of personality depending on the presence of stressors and the type of reinforcement. These results suggest that when negative reinforcement is used (i.e. stressor related to the task), the most fearful horses may be the best performers in the absence of stressors but the worst performers when stressors are present. On the contrary, when positive reinforcement is used, the most fearful horses appear to be consistently the worst performers, with and without exposure to stressors unrelated to the learning task. This study is the first to demonstrate in ungulates that stress affects learning performance differentially according to the type of reinforcement and in interaction with personality. It provides fundamental and applied perspectives in the understanding of the relationships between personality and training abilities.

## Introduction

How stress affects learning processes is a major question in behavioural science but has been extensively studied in a restricted array of species, mainly primates and rodents (synthesis: [1–4]). These studies aimed to determine whether stress affects cognitive performance and if it acts as either a stimulant or detriment for performance. One of the most common representations of this relationship is the well-known ‘Yerkes and Dodson law’ that has been encountered in various studies [1]. It describes an inverted U-shaped relationship between an individual’s state of stress and performance in a cognitive task. Nonetheless, the stimulant or deleterious effect of stress on cognitive performance can be determined by the complex interaction between the stress-induced release of various endogenous substances (neurotransmitters, neuropeptides and steroids) and the specificity of the brain regions implicated [5]. In addition, the effect of stress on cognitive performance also depends on how an individual may apprehend the apparent relationship between the stressor and the cognitive task. Especially stressors that are apparently external to the cognitive task appear to be more deleterious for learning performance than stressors that are apparently directly related to the cognitive task (also called intrinsic stressor, e.g. by using negative reinforcement) [3]. The reason may be that a state of stress is generally associated with a narrowing and reorientation of the attentional, motivational and cognitive processes toward the elements that appear threat-related but to the detriment of the unthreatening elements [6]. Therefore, if a stressor is confounded with the cognitive task, the performance could be enhanced. On the opposite, when a stressor is unrelated to the cognitive task, the cognitive performance is more likely to be impaired by the individual’s state of stress.

The main objective of the present study is to understand how stressors either related or unrelated to a learning task may affect learning performance in a non-rodent mammal. The secondary objective is to explore the possibility that the effects of stress on learning may vary across individuals. Indeed, variability of learning performance between individuals is a common phenomenon observed in a large number of studies and has been related to differences in personality (synthesis: [7–9]). In addition, a few studies have reported significant relationships between personality, cognition and stress [10–12]. For instance, students presenting with a highly anxious trait can be the best performers in a working memory task when performing under non-stressful conditions but are the worst performers when an external stressor is added (e.g. exposure to videogame competition [12]). Similarly in horses, fearfulness may have a deleterious effect on performance in working memory or learning tasks when stressors unrelated to the task are present (exposure to sudden and novel stimuli; [10,13,14]). Altogether, these studies suggest that the effect of a stressor on learning performance may be different according to personality, especially according to the traits related to emotional reactivity (e.g. fearfulness, *neophobia*, reactivity to suddenness). Therefore, we also explored the influence of personality on learning performance in our subjects.

To achieve this, we first assessed whether learning performance varies with the presence or absence of stressors unrelated to the task in horses performing an instrumental task based on positive or negative reinforcement. We consider that applying negative reinforcement is a form of intrinsic stressor, since it generates a potential source of stress directly related to the learning task. Four groups of horses were trained in an instrumental learning task while the exposure to stressors unrelated to the task (exposure to novel and sudden stimuli) and the type of reinforcement (positive or negative) was varied in order to compare their learning performances. Then, the relationship between personality and learning performance was investigated according to these two factors (stress and type of reinforcement). Horses were used in the present study, because a multidimensional model of personality has been well characterized in this species, with five independent dimensions found to be stable over time and across situations: fearfulness, gregariousness, reactivity to humans, level of locomotor activity and tactile sensitivity [15–18].

## Methods

### Subjects and general procedure

Subjects were 60 female Welsh horses aged 9 (5–12) years (median ± 1st and 3rd interquartile), bred at the French National Institute for Agricultural Research (INRA) of Nouzilly, France, and accustomed to being handled (regularly haltered and tethered). Before the experiment, the animals lived together outside in summer and inside in winter. During the experiment, they were housed in adjacent boxes with straw bedding (6 m × 4 m) in groups of three randomly mingled each day and spent 4 h daily in a large outdoor paddock. They received concentrated food (pellets) every evening, and hay and water were available ad libitum.

Horses were divided into four groups of 15 horses that were balanced according to age, sire and personality (see below for details regarding personality assessment):

1. *PR group*: horses were submitted to a task based on positive reinforcements (PR) without exposure to stressors unrelated to the task.
2. *PR+ES group*: horses were submitted to a task based on positive reinforcements (PR) combined with exposure to external stressors (ES) (i.e. unrelated to the task).
3. *NR group*: horses were submitted to a task based on negative reinforcements (NR) without exposure to stressors unrelated to the task.
4. *NR+ES group*: horses were submitted to a task based on negative reinforcements (NR) combined with exposure to external stressors (ES) (i.e. unrelated to the task).

### Learning procedure

In the days after the personality test, horses were required to perform an instrumental task that consisted of learning to walk into one of two compartments, which an experimenter pointed out, in order to get a food reward (PR and PR+ES groups) or to avoid unpleasant tactile stimulation (NR and NR+ES groups). The learning procedure was composed of different stages with increasing difficulty (i.e. cues given by the experimenter that were progressively more discreet). This task had been developed during preliminary studies so that learning could occur either with positive or negative reinforcement with an equivalent number of trials.

**Fig 1:**
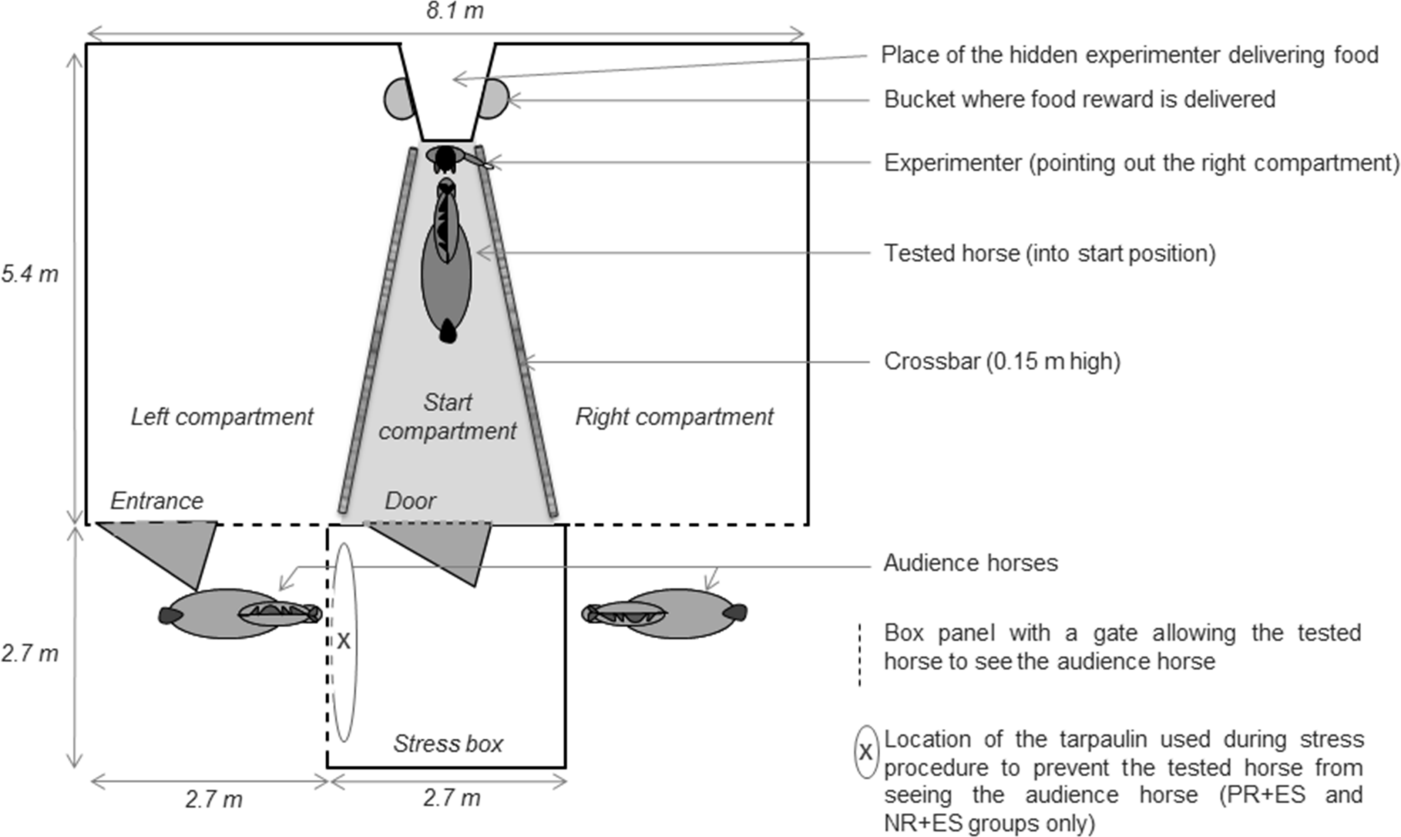
Experimental apparatus used for learning task.

Learning sessions were conducted in a test box composed of a start and a right and left compartments (Fig 1). The central start compartment was separated from the right and left compartments by wooden bars set on the floor (15 cm high). Two audience horses were placed outside of the test zone behind a panel with a gate and were visible to the tested horse to avoid stress induced by social isolation. In addition, there was an adjacent ‘stress box’ that could be visually isolated from the learning compartments and audience horses by closing a door. The use of the stress box will be detailed in the section devoted to the description of stress procedures.

Prior to the learning sessions, horses were submitted to familiarization sessions to give them the opportunity to fully explore the apparatus and become familiar with the feeding device (1 session/day). Each familiarization session consisted of a standardized sequence of four consecutive periods during which the horse alternated between free exploration of all the compartments and a guided exploration of each compartment and familiarization with the food delivery device. More specifically, during the first period, the horse was released into the box and was free to explore the entire test arena for 2 min (the stress box door was open). During the second period, the experimenter entered the test arena, put the horse into the stress box and closed the door for 1 min to familiarize the horse with being caged (an audience horse remained visible). During the third period, the experimenter opened the door and the horse was free to explore the entire test arena again for 1 min. Finally, during the last period, the horse was put into the stress box once again with the door closed for 1 min. To familiarize the horse with the food delivery procedure, a second experimenter hidden behind the box panels threw pellets (approximately 7 g) into one of two buckets (randomly right or left) twice per session. Thus, the horse could hear the food fall and eat it. This was done at the beginning of the first and third period when the horse had access to the entire arena. Familiarization sessions were repeated until the horse ate the pellets in less than 10 s twice during the session and exhibited neither vocalization nor excessive locomotion during a session (e.g. trotting or galloping), indicating completion of the familiarization criteria. All horses reached the familiarization criteria within two sessions, except for seven horses that needed at least three sessions.

**Fig 2:**
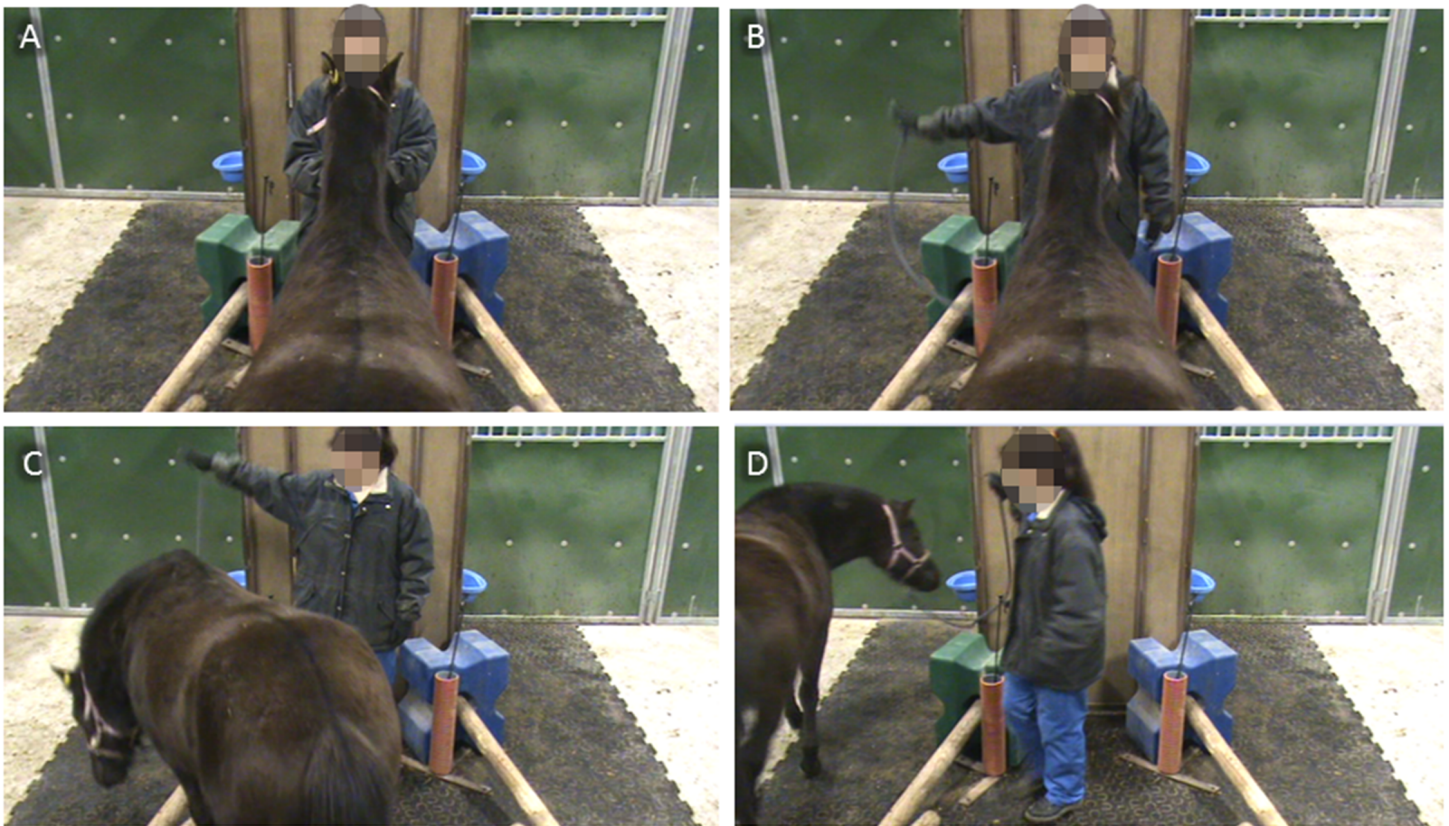
Sequential screenshots of a typical learning trial from a high-angle point of view. (A) Horse is ready in the starting position. (B) Experimenter is giving stage 1 indication toward the left compartment. (C) Horse is entering the left compartment. (D) Horse has successfully entered the left compartment and is going to receive food reward in the left bucket.

After having reached the familiarization criteria, the horses entered the learning phase where they were trained in the task until reaching all stages or a maximum of 120 trials divided into 8 sessions of 15 consecutive trials. Horses underwent one training session every two days. Before each trial, the horse was led with a lunge (*i*.*e*. the lead rope attached to the horse’s halter allowing a human to handle it) into the start compartment by an experimenter (a woman dressed with blue pants and a dark jacket). Once arrived at the start position, the experimenter held the horse while facing it with one hand on either side of the halter until the trial started (Figs 1 and 2). The trial started with the experimenter pointing out one of the two compartments with a dynamic gesture during 10 s (with movements going from the horse to the compartment). The side of the pointed compartment was chosen randomly at each trial, with a 50/50 chance but no more than two consecutive trials using the same compartment. The type of gesture used by the experimenter depended on the learning stage (detailed gestures are presented in Table 1), ranging from the easiest gesture to detect (stage 1) to the hardest (stage 4). Each horse was submitted to a new stage as soon as it performed 4 out of 5 successful consecutive trials in a given stage. A trial during which the horse directly went into the pointed compartment (i.e. without going into the wrong compartment first) in less than 5 s with all four legs crossing the wooden bar was considered as a success (same criterion for all groups).

**Table 1:** Trial procedure for each learning stage.

Stage (by growing difficulty)	Description of the procedure
1	The experimenter points out the compartment for 10 s with his entire arm extended horizontally by making dynamic movements from the horse to the compartment while holding the lunge in his hand in such a way that with each movement, the lunge is slightly tautened toward the compartment.
2	The experimenter makes the same action as during stage 1 for 10 s except the lunge is not present and consequently its weight does not exert any physical pressure on the horse.
3	The experimenter points out the compartment for 10 s with his forearm extended horizontally by making dynamic movements from the horse (without any physical contact) to the compartment.
4	The experimenter performs the same action as during stage 3 for 10 s except he only uses his hand to point out the compartment.

If the horse did not go into the compartment pointed out after 10 s, the experimenter encouraged the horse by guiding it during an additional period of maximum 10 s. The objective was to give additional information to help the horse associate the experimenter’s cue to the requested behaviour (i.e. going to the pointed compartment). This guidance consisted of emphasizing the experimenter’s gesture with the addition of a movement from the chest (stages 1 and 2) or by using a previously learned indication (stage 3: pointing at the compartment with the entire arm, stage 4: pointing at the compartment with the forearm). If the horse did not enter the compartment after 10 s of guidance, or if it went into the wrong compartment at any time, the experimenter led it to the appropriate compartment with a lunge. For both PR groups, as soon as the horse moved either spontaneously or with guidance into the pointed compartment, it immediately received a handful of pellets (approximately 7 g) delivered into the bucket by an experimenter hidden behind the panel (into the bucket of the pointed compartment). For both NR groups, aversive tactile stimulations started 5 s after the beginning of the trial, or as soon as the horse went into the wrong compartment, and were stopped as soon as it entered into the pointed compartment. Stimulations consisted of patting on the flank with a stick to induce an unpleasant stimulation (the objective was to encourage the horse to move by tickling a sensitive body part but not to induce a painful sensation). The tactile stimulation was applied on the flank at the opposite side from the pointed compartment. To avoid any confusion, it is important to explain the use of the term “negative reinforcement” in the present case. When horses entered into the pointed compartment before the beginning of the unpleasant stimulation, it is known as avoidance learning since the response prevents the stimulation. When horses entered into the pointed compartment after the beginning of the unpleasant stimulation, it is known as escape learning since the response stops the outcome (see also [19]).

Due to practical reasons, two different women took on the role of the experimenter directing the horse (4 sessions each, alternatively). The sessions conducted by each experimenter occurred within the same time-course for every horse. To avoid any bias, the experimenters were previously trained to standardize their gestures and they wore the same outfits.

The first measurement of learning performance consisted of the number of trials needed to reach the stage 1 criterion (five consecutive successful trials, i.e. going into the pointed compartment in less than 5 s), which corresponds to the learning speed of the task’s basic rule. The second measurement was the number of stages reached, which corresponds to the maximum level of learning an animal can achieve during the procedure.

### Stress procedure and measurements

Before each block of five learning trials (i.e. three times per session), all the horses were led and released by a second experimenter into the adjacent stress box for 30 s. For the groups not exposed to stressors unrelated to the learning task (PR and NR), no particular event occurred during these 30 s and the horses could see an audience horse. By contrast, horses from the groups exposed to stressors unrelated to the learning task (PR+ES and NR+ES) could not see the audience horse because a tarpaulin covered the wall. In addition, these horses were submitted to three sudden events separated by 10-s intervals without seeing the experimenters: emission of loud noises (e.g. dog barks, bell ringing, people talking loudly), surprise movements (shaking of the tarpaulin, water or air jet emitted toward the horse) and introduction of an unfamiliar object (e.g. colourful cardboard box, colourful balloons; for more details see [13]). The sequence of three stressors changed between each episode, but was always the same between the horses. Immediately afterwards, the horses were led into the start compartment to begin a new learning trial.

Behavioural observations were made during the 10-s intervals when the horses were held in their start position before the beginning of each trial. We recorded the number of times the horse sniffed or nibbled the experimenter, glanced at the audience horse, exhibited an alert posture, made blowing sounds, showed startle reactions, pointed the ears backward, explored the environment (sniffing or nibbling the floor), defecated or crossed the wooden bar.

At sessions 1 and 3, two salivary samples were collected before and after the learning procedure using cotton buds. Therefore, the first sample was collected before any learning or stress procedure (in the home stable, just before the horse was brought to the learning area), and the second sample was collected just after the whole session (including 15 learning trials and the three stress exposures), immediately after the horse was brought back to its home stable. These two sessions were chosen because they covered a period during which all the horses underwent learning sessions. At session 4, some horses had indeed reached the learning criteria for all the stages and were not trained anymore. The cotton buds with the samples were then centrifuged at 3000 g for 20 min at 4° C and the saliva stored at -20° C until analysis. Saliva was collected and cortisol was measured in 20 μl samples by using a luminescence immunoassay kit (LIA, IBL, Hamburg, Germany). The measurements were performed without replicates in a single assay. The intra-assay coefficients of variation were 4.8% and 4.1% at 1.8 ng/ml and 9.7 ng/ml, respectively. The assay sensitivity was 0.25 ng/ml. We compared the increase in salivary cortisol concentration between groups (i.e. increase = concentration after session–concentration before session).

### Personality tests

The week before the learning experiment, we submitted each horse to an array of behavioural tests to assess the five independent dimensions of personality previously shown to be stable across situations and over time: fearfulness [16], gregariousness [17], reactivity to humans [15], level of locomotor activity [20] and tactile sensitivity [18]. This array of tests is standardised and has been designed to measure specific personality parameters. The stability and reliability of these parameters have been proven by the work of Lansade and collaborators [15–18,20,21].

Personality tests were conducted in a box (2.7 m × 8.1 m) and several behavioural parameters were recorded (for details concerning procedure see [13,22]). An audience horse, the same throughout the experiments, unfamiliar to the tested horses and chosen for its quietness, was tied up outside the box visible to the tested horse to avoid a social isolation context. After a familiarization phase during which the tested horse was free to move in the box for 5 minutes, the tests were conducted consecutively in the following order:

1. Passive human test (reactivity to humans): The experimenter, unfamiliar to the horses, entered the box and stayed motionless for 180 s. The number of contacts with the experimenter was recorded (sniffing or nibbling, for descriptions see [23,24]).
2. Tactile sensitivity test (sensory sensitivity): The experimenter successively applied four von Frey filaments (0.008 g, 0.02 g, 1 g and 300 g, Stoelting, IL, USA) perpendicularly to the base of the horse’s wither. The number of times the horse reacted to the filaments, indicated by trembling of the platysma muscle, was recorded.
3. Novel object test (fearfulness): An object, unknown to the horse, was placed in the box for 180 s (suspended along the wall at a height of 1.4 m). The number of contacts (sniffing or nibbling, indicating an absence of fear) and the number of glances (indicating the presence of fear, for a full description see [16]) at the novel object were recorded.
4. Social isolation test (gregariousness): The audience horse was removed from sight and vocalizations and the number of neighs of the tested horse were recorded for 90 s (neighs are the most representative indicators of gregariousness according to [17]).
5. Novel area test (fearfulness): In order to get access to a familiar bucket containing pellets, the environment was arranged such that the tested horse had to first cross a colourful carpet. The time the horse took until eating on the other side of the carpet was recorded, along with the number of glances at the carpet (both indicating the presence of fear).
6. Surprise test (fearfulness): An umbrella was opened in front of the animal 3 s after it started eating from the bucket of pellets. The response was assessed by measuring the intensity of the startle response that was quantified on the base of the observation criteria established by Lansade [20].

In order to assess the locomotor activity dimension, the box was virtually divided into 6 areas of equal size. The number of areas crossed during the familiarization phase, passive human test, novel object test and social isolation test were recorded. In addition, to assess fearfulness, the number of blowing behaviours was continuously recorded (each blowing sound was recorded as a behavioural item).

### Statistical analyses

The XLSTAT software (Addinsoft Software, Paris, France) was used to analyse the data. Comparisons between groups were made using Kruskal-Wallis tests followed by Dunn tests by pair in case of significance or tendency. Spearman rank correlations were calculated between the parameters recorded during the personality tests and learning performance. The behaviour observed during the inter-trial intervals was analysed only during the first three learning sessions by summing the number of observed behaviours in the entire period. These sessions were chosen because they covered the period during which all the horses were present during learning. Results are presented as median value and interquartile range. The level of statistical significance was set at *P* ≤ 0.05 and the level of tendency at *P* ≤ 0.10.

### Ethical note

The experiment was conducted under a license from the French Ministry of Agriculture (no. 37–125). Animal care and experimental treatments complied with the guidelines of the French Ministry of Agriculture for animal experimentation and European regulations on animal experimentation (86/609/EEC) and were performed in accordance with the local animal regulation (authorization N° 006352 of the French Ministry of Agriculture in accordance with EEC directive). The protocol has been submitted to an ethics committee (CEEA VdL, France) but the committee points out that it cannot evaluate the present protocol since no intervention on the animals occurs (rules = no ethical statement is needed for this type of protocol). At the end of the experiment, the animals returned to normal breeding at the INRA station. No signs of injury or pain were observed during or after the experiments. The acute stressors were inspired by real stressors often encountered by domestic animals, which consist of short-term social isolation lasting a few seconds combined with novel and sudden stimuli or events. The horses lived in social groups and were taken to a paddock daily. The horses were not food restricted during the entire experimental period.

## Results

### Effects of type of reinforcement and exposure to stressors unrelated to the learning task

**Fig 3:**
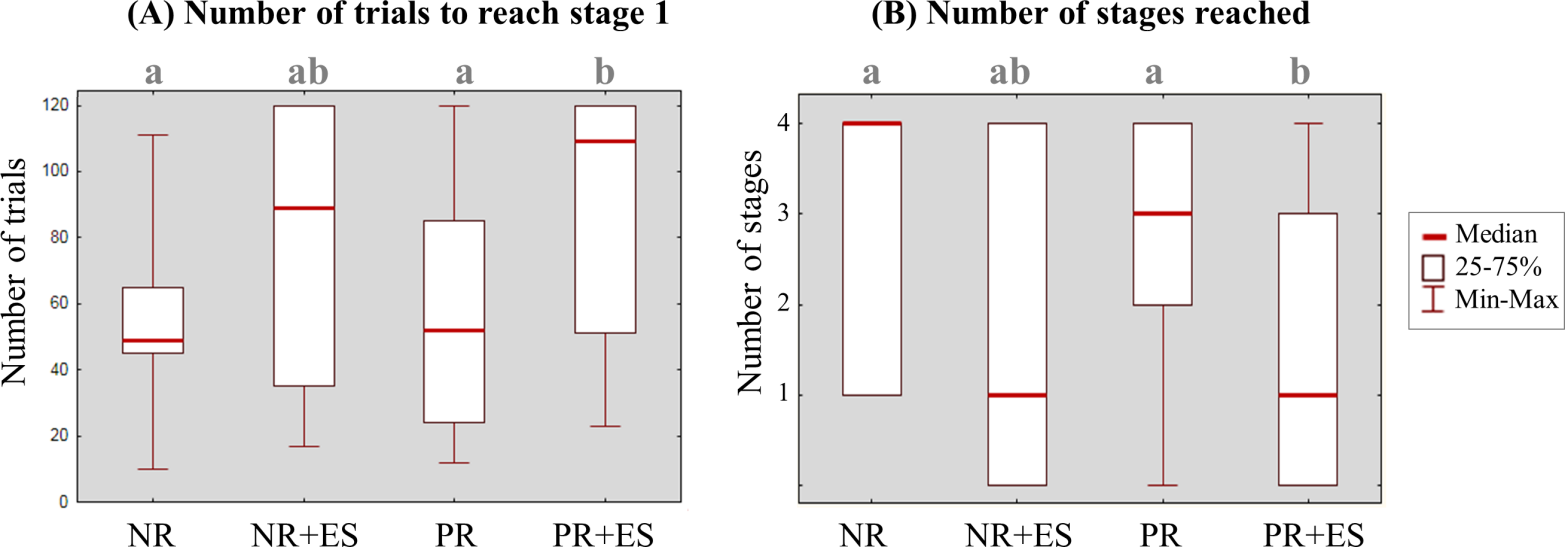
**Number of trials needed to reach the stage 1 criterion (A) and number of stages reached (B).** A high number of stages reached and a low number of trials to reach stage 1 are both indicative of a high learning performance level. PR: positive reinforcement, NR: negative reinforcement, ES: exposure to extrinsic stressor (i.e. unrelated to the learning task). Values are expressed as median and interquartile range; a vs. b, *P* < 0.05, Kruskal-Wallis tests, *df* = 3, followed by Dunn tests.

In the absence of stressors unrelated to the learning task, the learning performance (number of trials to reach stage 1 and number of stages reached) did not differ between the two types of reinforcement (Kruskal-Wallis _trials to reach stage 1_: K = 7.07, P = 0.07, Kruskal-Wallis _trials to reach stage 1_: K = 8.68, P = 0.03, Dunn test PR vs. NR, *P*_*trials* to reach stage 1_ = 0.80, *P*_*stages reached*_ = 0.78, Fig 3A and 3B). However, when stressors unrelated to the learning task were added, learning performance significantly decreased with positive reinforcement and tended to decrease with negative reinforcement (Dunn test PR vs. PR+ES: *P*_*trials* to reach stage 1_ = 0.03, *P*_*stages reached*_ = 0.04; Dunn test NR vs. NR+ES: *P*_*trials* to reach stage 1_ = 0.06, *P*_*stages reached*_ = NS; Fig 3A and 3B).

In the absence of stressors unrelated to the learning task, the observed behaviours did not differ between reinforcement types (Table 2, comparison between PR and NR). However, when stressors unrelated to the task were added, horses exhibited significantly more alert postures, startle reactions and glancing at the audience horse under both reinforcements contingencies; in the NR group, they also showed more ears pointing backward and blowing (Table 2, comparison between PR vs. PR+ES or NR vs. NR+ES). No significant differences were observed between the four groups in the number of times the horses sniffed or nibbled the experimenter, explored the environment, defecated or crossed the wooden bar.

**Table 2:** Median (1st–3rd interquartiles) of behavioural variables exhibited by each experimental group during the first three learning sessions.

	Experimental group				
Behaviour	NR	NR+ES	PR	PR+ES	Kruskal-Wallis values
Alert	1 (0.5–3.5) ^a^	11 (4–14) ^b^	2 (0–4.5) ^a^	6 (4.5–11) ^b^	K = 19.50, P<0.001
Startle reactions	0 (0–0) ^a^	1 (1–3) ^b^	0 (0–0) ^a^	2 (0.5–3.5) ^b^	K = 26.30, P<0.001
Glancing at audience horse	7 (3.5–12.5) ^a^	20 (13.5–27.5) ^b^	4 (2–9) ^a^	19 (15–23.5) ^b^	K = 16.32, P = 0.001
Ears backward	2 (0.5–11) ^a^	10 (7–19.5) ^b^	1 (0.5–7) ^a^	5 (2.5–12) ^ab^	K = 11.22, P = 0.01
Blowing	1 (0–5) ^a^	6 (2.5–9) ^b^	0 (0–2) ^a^	1 (0.5–4.5) ^ab^	K = 10.78, P = 0.01

PR: positive reinforcement, NR: negative reinforcement, +ES: exposure to extrinsic stressor (i.e. unrelated to the task).

Kruskal-Wallis tests, *df* = 3, followed by Dunn tests.

For each behaviour, different letters indicate statistical differences between experimental groups (a vs. b, Dunn tests, *P* < 0.05) and identical letters indicate an absence of statistical difference (P > 0.05).

Only behaviours differing between groups are shown in this table.

In the absence of stressors unrelated to the task, the increase in salivary cortisol concentrations did not differ between groups, neither in session 1 nor 3. When stressors unrelated to the task were added, the increase in concentration was significantly higher but only in the case of positive reinforcement (Dunn test PR vs. PR+ES: *P*_*Session 1*_ *<* 0.05, *P*_*Session 3*_ *<* 0.01; Table 3).

**Table 3:** Median (1st–3rd interquartiles) of increases in salivary cortisol concentration at learning sessions 1 and 3 (differences in ng/ml).

	Experimental group			
Increase in salivary cortisol	NR	NR+ES	PR	PR+ES
Session 1	0.13 (-0.03–0.26) a	0.28 (0.07–0.61) ab	0.05 (-0.29–0.41) a	0.40 (0.34–0.54) b
Session 3	0.11 (-0.05–0.14) a	0.10 (-0.08–0.26) a	-0.04 (-0.16–0.18) a	0.53 (0.15–0.93) b

PR: positive reinforcement, NR: negative reinforcement, +ES: exposure to extrinsic stressor (i.e. unrelated to the task).

Kruskal-Wallis tests, *df* = 3, followed by Dunn tests. Kruskal-Wallis tests: K = 7.36, P = 0.06; Session 3: K = 8.83, P = 0.03. Different letters indicate significant differences between experimental groups (a vs. b, Dunn tests, *P* < 0.05).

### Relationships between personality and learning performance

Horses learning with negative reinforcement without stressors unrelated to the task (NR) that required the shortest time to reach the stage 1 criterion (i.e. the best performers among the NR horses) were the most fearful and active (correlation between the number of trials to reach stage 1 and *a)* intensity of the startle response during the surprise test: *r*_*s*_ = -0.57, *P* = 0.03; and *b)* number of areas crossed: *r*_*s*_ = -0.61, *P* = 0.02).

Horses learning with negative reinforcement and exposure to extrinsic stress (NR+ES) that reached the highest number of stages (i.e. the best performers among the NR+ES horses) were the least fearful and active (correlations between the number of stages reached and *a)* number of glancing at the novel area: *r*_*s*_ = 0.54, *P* = 0.04; *b)* total number of blowing: *r*_*s*_ = 0.55, *P* = 0.03; *c)* latency before eating during the novel area test: *r*_*s*_ = 0.48, *P =* 0.07 (tendency); *d)* number of contacts with the novel object: *r*_*s*_ = -0.45, *P* = 0.09 (tendency); and *e)* number of areas crossed: *r*_*s*_ = -0.54, *P* = 0.04). In addition, the fastest horses to reach the stage 1 criterion in the NR+ES group (the best performers) were also the least fearful, the closest to humans and the most active (correlations between the number of trials to reach the stage 1 criterion and *a)* number of contacts with the novel object: *r*_*s*_ = -0.52, *P* = 0.04; *b)* number of contacts with the passive human: *r*_*s*_ = -0.57, *P* = 0.03; and *c)* number of areas crossed: *r*_*s*_ = -0.51, *P* = 0.05).

By contrast, only one negative correlation between a variable related to fearfulness and the level of learning performance was found in each group of horses with positive reinforcement (PR and PR+ES). This correlation indicated that horses reaching the highest number of stages (i.e. the best performers among PR and PR+ES horses respectively) were the least fearful in both groups (correlations between the number of stages reached and *a)* the number of glancing at the novel area in PR group: *r*_*s*_ = -0.51, *P* = 0.05; and *b)* the number of glancing at the novel object in PR+ES group: *r*_*s*_ = -0.57, *P* = 0.03).

## Discussion

Our study shows that exposure to stressors before testing has a negative effect on learning performance, mainly under conditions of positive reinforcement. In addition, learning performance appears to be differentially related to personality according to the type of reinforcement and the presence of extrinsic stress. In the absence of stressors unrelated to the task, the most fearful horses were the best performers when they learned with negative reinforcement but the worst when they learned with positive reinforcement. When stressors unrelated to the task were applied, the most fearful horses were consistently the worst performers, particularly with negative reinforcement learning.

### Stressors unrelated to the task impair learning performance according to the type of reinforcement

In the absence of stressors unrelated to the task, we did not observe any effect of reinforcement type on learning performance and behavioural or physiological parameters. However, in the presence of stressors unrelated to the task, learning performances decreased or tended to decrease in both groups (NR+ES and PR+ES). Impairment of attention induced by the stressors could be involved [1]. Horses exposed to stressors unrelated to the task displayed more startled reactions and alert behaviours oriented outside of the learning task and directed at the audience horse than horses not exposed to stressors unrelated to the task, suggesting a decrease in attention toward the learning task itself, as observed previously [13]. Interestingly, this learning deficit appeared to be smaller in horses learning with negative reinforcement than in horses learning with positive reinforcement. This result could be explained by the fact that negative reinforcement in itself could be considered a stressor directly related to the cognitive task, known to alleviate learning performance by focusing attention toward the learning task ([1,3], e.g. [25]). This focus of attention toward the task could have counterbalanced the impact of the stressors unrelated to the task in the NR group. In addition, acute stress is known to decrease food motivation [26–28]. An adaptative response to stress consists for an organism to prepare itself to react to this threat, generally based on a fight or flight response. Escaping becomes then the priority other eating. Within a behaviour analytic framework, fear could serve as a potential establishing operation [29], increasing the effectiveness of escaping as a reinforcer. In contrast, that fear could serve as an abolishing operation for food as a reinforcer. Therefore, an impaired motivation for food rewards may also explain why learning performance was less impaired with negative than with positive reinforcement (i.e. food reward).

Different patterns of behavioural and physiological responses between PR+ES and NR+ES horses were observed but did not provide an explanation for which group was more stressed. Elevated salivary cortisol levels indicate that the stress level might have been higher in horses learning with positive reinforcement, since PR+ES horses showed increased cortisol levels. However, NR+ES horses but not PR+ES horses showed more ears pointing backward and blowing, which might both indicate discomfort or stress [30–32]. One reason that may explain those differences is the history with escaping a stressor that changes according to the reinforcement type. Indeed, during each learning session, NR+ES experienced several times the avoidance of a negative outcome (i.e. tactile stimulation) by going actively into a safe compartment (i.e. pointed compartment). Experiencing this active escaping repeatedly may help reduce cortisol, whereas PR+ES horses did not experience such active process to cope with stress. However, even though the present results suggest that different patterns of stress response may emerge, they do not allow establishing clearly which reinforcement type was more stressful, and further investigations are needed to improve our knowledge of discomfort and stress measurements in horses.

### Importance of personality in relationship between stress and learning performance

Our study reveals the existence of relationships between personality and learning performance. However, because personality is only one factor among others of predisposition of the learning performances, these relationships are tenuous. That explains why, at a statistical level of 5% and with only 15 animals per group, only a few variables of personality are significantly correlated with learning performance. However, what is interesting is that that these relationships vary with the presence of stressors unrelated to the task and type of reinforcement, especially for the dimension of fearfulness. In the absence of stressors unrelated to the task, the most fearful horses appear to be the best performers with negative reinforcement learning (NR group) but the worst when they had to learn with positive reinforcement learning (PR group). The fact that fearfulness might be advantageous when animals learn with negative reinforcement but disadvantageous when they learn with positive reinforcement is in agreement with previous experiments (active avoidance in guppies [33], instrumental task in horses [22,34,35], instrumental task of discrimination in ravens [36], instrumental cooperative task in rooks [37]). It is possible that the positive effect of negative reinforcement (considered as stressor) on performance, through the focus of attention toward the task, might have been accentuated in fearful individuals and could explain their improved performance in the absence of stressors unrelated to the task. By contrast, there were no stressors related to the learning task in the PR group, and the fearful horses might have been more easily distracted by the external environment. When stressors unrelated to the task were added, learning performance of the most fearful horses appeared to be consistently impaired, independent from the type of reinforcement (PR+ES and NR+ES groups). The disruption of cognitive and attentional processes caused by the stressors unrelated to the task is likely to be more pronounced in fearful individuals who react more strongly to various forms of stressors (e.g. [37], synthesis: [38,39]).

Finally, our study indicates that other dimensions of personality may also influence learning performance. Locomotor activity was positively related to performance in negative reinforcement learning, independent from exposure to stressors unrelated to the task. We hypothesize that locomotor activity may broadly reflect a tendency to initiate actions, since we previously observed such a positive effect with learning tasks requiring a displacement of body position similar to the present task [22], but also when requiring different types of movement (touching an object with the nose: [13]). In addition, reactivity to humans was negatively correlated with learning performance in the NR+ES group only. The horses less frightened by humans might have been less affected by the stressors unrelated to the task because they were more attentive to human cues.

We have to mention again that with 15 animals per group, only a few significant correlations between variables of personality and learning performance were revealed. To reveal the whole influence of personality on learning abilities, it would be ideal to perform this kind of studies on a larger amount of subjects, maybe several hundreds. Unfortunately, it is quite impossible to test this number of subject, within the equine species. However, we have to underline the fact that, taken together, all the experiments carried out on horses reveal consistent links between personality and learning abilities [10,13,14,22,40]. It would mean that these correlations are not obtained by chance, but more likely reflect a real influence of personality.

### Conclusion

The present study contributes to a better understanding of the influence of stress on learning performance by showing the importance of the nature of the stress (related or unrelated to the task) and personality. Our results also provide important clues to more personalized training according to each animal’s needs. Indeed, the present study shows that positive and negative reinforcement may lead to equivalent learning performance in the absence of stressors unrelated to the task. Considering that previous studies highlighted long-lasting promising effects of positive reinforcement on training, welfare and relationships with humans [32,41–46], our results provide additional evidence for promoting the use of positive reinforcement in horse training, while the use of negative reinforcement still dominates traditional training methods [47,48]. However, the present results also show that the use of food reward as reinforcement may not be adequate in stressful conditions. Indeed, the loss of food motivation induced by stress could render the reinforcement inefficient and constitute an additional source of stress. In addition, our analyses including personality suggest that stress is a key factor in understanding how animals differ in learning performance according to their personality. Predicting how fearful animals react when they face a learning challenge therefore not only requires an evaluation of stress level but also of the nature of the stress (related or unrelated to the task) and their possible interactions. Future investigations are needed to define more precisely when the switch from favourable to unfavourable conditions for learning occurs according to the type and the intensity of a stressor and how it relates to personality.

## Supporting information

S1 File Data set. (XLSX)

S2 File Ethical statement. (PDF)
