# Supplementary material for: Stress affects instrumental learning based on positive or negative reinforcement in interaction with personality in domestic horses
Source: PLoS One. 2017 May 5;12(5):e0170783. doi: 10.1371/journal.pone.0170783 (PMC5419560; doi:10.1371/journal.pone.0170783)
Supplement: S2 File — (PDF) [file pone.0170783.s002.pdf]

Comité d'éthique
pour l'expérimentation animale

Comité d'éthique de Val de Loire (CEEA VdL)

Enregistré auprès du Comité National de Réflexion
Ethique sur l'Expérimentation Animale
Sous le numéro 19

English translation of the CEEA VdL statement (local Ethical Committee) regarding our procedure
Original document can be finding on page 2

File number: 2012-12-23
Name: Léa Lansade
Title: Study of the relationships between temperament, stress and learning in horses

Dear Madam,

We received your document regarding your protocol "*Study of the relationships between temperament, stress and learning in horses*" that has been submitted to *the Val de Loire Ethical Committee (CEEA VdL).*

The *CEEA VdL* points out that it cannot evaluate the present protocol since no intervention on the animals occurs (Rules = no ethical statement is needed for this type of protocol). However, the President of the CEEA VdL informs you that, in certain cases, it is able to read the protocols, assign a positive recommendation and sign them in order to attribute a registration number.

The *CEEA VdL* attributes a positive recommendation.

Yvelise Fillet Coordinatrice
✆ 06 62 00 92 48
Courriel : ceeavdl@univ-tours.fr

CHRU Bretonneau - Bât B1a - 1er étage
2, Bd Tonnellé - 37044 Tours Cedex 9

Comité d'éthique
pour l'expérimentation animale
Comité d'éthique de Val de Loire (CEEA VdL)

Tours le, 14 décembre 2012

N° Dossier : 2012-12-23
Nom : Léa Lansade
Intitulé : Etude des relations entre tempérament, stress et apprentissage chez le cheval

Madame,

Nous avons bien reçu votre document de saisine relatif à votre protocole " Etude des relations entre tempérament, stress et apprentissage chez le cheval ", document soumis au Comité d'Éthique Val de Loire.

Le CEEA VdL précise qu'il n'évalue pas le document puisque qu'il n'y a aucune intervention sur les animaux vivants (Réglementation = pas d'évaluation éthique nécessaire pour ce type de protocole) mais informe que la Présidence du CEEA VdL, dans certains cas, pourra lire ces protocoles, donner un avis favorable et les signer afin d'attribuer un numéro d'enregistrement.

Le CEEA VdL émet un avis favorable.

Très cordialement,

Sophie Fouchécourt — Michèle de Monte

Yvelise Fillet Coordinatrice
✆ 06 62 00 92 48
Courriel : ceeavdl@univ-tours.fr
CHRU Bretonneau - Bât B1a - 1er étage
2, Bd Tonnellé - 37044 Tours Cedex 9
